# CYP and SXR gene polymorphisms influence in opposite ways acute rejection rate in pediatric patients with renal transplant

**DOI:** 10.1186/s12887-020-02152-3

**Published:** 2020-05-25

**Authors:** Stefano Turolo, Alberto Edefonti, Luciana Ghio, Sara Testa, William Morello, Giovanni Montini

**Affiliations:** 1 Fondazione IRCCS Cà Granda Ospedale Maggiore Policlinico UOC Nefrologia Dialisi e Trapianto pediatrico, Via della, Commenda 9, 20122 Milan, Italy; 2 Department of Clinical Sciences and Community Health, University of Milan, Milan, Italy

**Keywords:** Kidney transplantation, Acute rejection, *SXR*, *CYP*, Tacrolimus, Pharmacogenomics

## Abstract

**Background:**

We evaluated the role of *CYP3A5, ABCB1* and *SXR* gene polymorphisms in the occurrence of acute kidney rejection in a cohort of pediatric renal transplant recipients.

**Methods:**

Forty-nine patients were genotyped for *CYP3A5, ABCB1* and *SXR* polymorphisms and evaluated with tacrolimus through levels in a retrospective monocenter study.

**Results:**

Patients with the A allele of *CYP3A5* treated with tacrolimus had a higher risk of acute rejection than those without the A allele, while patients carrying the homozygous GG variant for *SXR A7635GG* did not show any episode of acute rejection.

**Conclusion:**

Genetic analysis of polymorphisms implicated in drug metabolism and tacrolimus trough levels may help to forecast the risk of acute rejection and individualize drug dosage in children undergoing renal transplantation.

## Background

Acute rejection occurs in up to 10–15% of patients during the first year following kidney transplantation [1], and is associated with long-term allograft dysfunction. The immune response directed against the graft is the result of either acute cellular rejection, due to a T-cell-dependent process, or acute humoral rejection, generated by B-cells [2–6]. Several factors influence the occurrence of acute rejection: recipient clinical and immunological characteristics (particularly HLA donor/recipient mismatch), donor clinical and biochemical data, and transplant-related factors [7]. Potent immunosuppressive agents have significantly increased the short- and long-term allograft and patient survival [8, 9], but inadequate doses of immunosuppressive drugs may be found in clinical practice, leading to clinical or subclinical reactivation of the immune system.

Tacrolimus is the main calcineurin inhibitor used in kidney transplantation in high-income countries [10]. It is metabolized by cytochrome P-450, encoded by the CYP genes cluster. It is well known that polymorphisms of the intracellular metabolizer enzyme CYP and the trans-membrane transport protein ABCB1 may influence enzymatic intracellular activity, modifying drugs metabolism [11–19]. Patients with the A allele on CYP3A5*3 need to double the dose of tacrolimus in order to reach therapeutic blood concentration [20]. Additionally, *ABCB1* polymorphisms may affect, either positively or negatively, tacrolimus metabolism [21], even if to a lesser extent. The expression of both *CYP* and *ABCB1* genes is regulated by the intracellular receptor SXR [22, 23], which, after activation, makes up a heterodimer with various molecules to act as a transcriptional activator [23]. It has been reported that *SXR A7635G*, an intronic single nucleotide polymorphism (SNP), is able to increase tacrolimus clearance [24, 25]. On the other hand, studies in kidney transplant recipients showed no effect of this SNP on tacrolimus blood concentration [26, 27].

More recently, tacrolimus through levels were correlated with the risk of acute rejection during the first-year post transplantation [28]. However, a study of genes polymorphisms involved in the calcineurin pathway did not find any positive correlation between the main SNPs and acute rejection rate [29].

The aim of this retrospective study was to evaluate the role of *CYP3A5, ABCB1* and *SXR* polymorphisms on tacrolimus through levels and acute rejection rate in a paediatric population during the first year following kidney transplantation.

## Methods

### Patients

We analyzed the data of 49 children transplanted between January 2000 and December 2010 in a single Pediatric Nephrology unit. Inclusion criteria were: age between 1 and 18 year old, clinical and laboratory follow up for at least 1 year, data on blood trough levels of Tacrolimus at 1 week, 1,3,6 months and 1 year and data on *CYP3A5, ABCB1* and *SXR* polymorphisms. Exclusion criteria were simultaneous liver-kidney transplantation.

### Clinical data

Tacrolimus was administered at a dose of 0.3 mg/kg/day in order to achieve trough blood levels (C0) of 10–20 ng/ml during the first two post-transplant months and 5–10 ng/ml thereafter. The calcineurin inhibitor was administered in combination with mycophenolate mofetil at a starting dose of 600–800 mg/m^2^ /day, aiming for a C0 of 1.5–3 μg/ml.

Steroids were given intravenously (10–15 mg/kg/day) for the first two postoperative days and then orally at a dose of 1 mg/kg/day, which was gradually tapered to 0.125 mg/kg/day by 6 months after transplantation.

The diagnosis of acute rejection was made on the clinical and laboratory grounds, increase of more than 20% of serum creatinine, appearance of proteinuria, and reduction of urinary output. The diagnosis was confirmed by renal biopsy, according to Banff criteria [30, 31].

HLA mismatching, tacrolimus through blood levels and gene polymorphisms of *CYP3A5, ABCB1* and *SXR* were analysed as risk factors of acute rejection rate.

As regards tacrolimus, whole blood sampling was performed at 6, 30, 60, 180 and 360 days after transplantation and the following pharmacological parameters were assessed: tacrolimus trough blood level (C0: ng/ml), daily dose per body weight (mg/kg) and dose-normalized trough level (C0/dose/kg BW). Tacrolimus blood concentration was measured using Syva® EMIT (Dade Behring, Eschborn, Germany).

### Genotyping

As regards genotyping of *CYP3A5, ABCB1* and *SXR* polymorphisms 500 μl of whole blood were collected during routine ambulatory control.

DNA extraction was performed by extractor Fuji QuickGene-810 (Fujifilm, Tokyo, Japan), PCR was carried out in 20 μl of a solution containing 2 μl of 10 x PCR Gold Buffer, 2 mM of MgCl_2_ (Applied Biosystem, Foster City, CA, USA), 80 μM each of dNTPs (Euroclone, Pero, Milan, Italy), 50 pmol each of primers for CYP3A and ABCB1 as previous described [32], 50 ng of genomic DNA and 0.6 U of AmpliTaq Gold (Applied Biosystem, Foster City, CA USA). For the polymorphism of *SXR A7635G* and *SXR –200 GAGAAG/−* (rs3842689) we used the following primers: *SXR A7635G* forward 3′- TGG ATG CCA AGC TCA GTGG − 5′; reverse 3′- CAG CAG CCA TCC CAT AAT CC − 5′; for *SXR* rs3842689 we used the following primers pair: forward 3′-CTG ATG CTC TCT GGT CCT GC − 5′, reverse 3′-TGC CTG CTA TAG CTG ATT CAT TG-5′ with a melt temperature of 60 °C for both polymorphisms..

The template was purified by liquid handling Biomek® 3000 (Beckman Coulter, CA, USA) using a magnetic particles system (Agencourt/Beckman Coulter, CA, USA). The single DNA strand was amplified by BigDye® 3.1 (Applied Biosystems, Foster City, CA USA) and then sequenced by a 3130xl Genetic Analyzer (Applied Biosystems/Hitachi, Foster City, CA USA).

### Statistical analysis

Data were analyzed with Mann Whitney test for pharmacological data, and Fisher exact test for the acute rejection data, a *p*-value < of 0.05 was considered significative. All analyses ware performed with SPSS software (IBM).

## Results

Forty-nine pediatric patients who received a kidney transplant between January 2000 and December 2010, from either deceased (44) or living (5) donors, and who were treated with an immunosuppressive protocol including tacrolimus and with a complete set of tacrolimus trough blood levels and pharmacogenomic data were available for evaluation. Their demographic and clinical characteristics are shown in Table 1.

**Table 1:** Demographic data of the pediatric renal transplant recipients

Variable	Value
Male/female	28/21 (*n* = 49)
Age (Mean ± SD)	15.6 ± 6.1 (3 under 6 years of age)
Weight (Mean ± SD)	
Day 7:	44.69 ± 17.87 kg
Day 30:	44.86 ± 18.00 kg
Day 90:	47.76 ± 18.7 kg
Day 180:	49.31 ± 18.66 kg
Day 360:	50.72 ± 18.09 kg
Ethnicity:	
Caucasian:	46 (94.0%)
Hispanic:	2 (4.0%)
North Africa:	1 (2.0%)
Primary renal disease	
CAKUT	16 (32.6%)
Glomerulonephritis	12 (24.5%)
Vasculitis	6 (12.2%)
Tubulopathy	6 (12.2%)
Other	9 (18.3%)

CAKUT = Congenital Abnormalities of the Kidneys and the Urinary Tract

### HLA mismatch

Eight patients had one episode of acute rejection during the first year post transplantation.

Recipients’ HLA matches and mismatches are shown in Table 2. The majority of the patients (27/49) matched the donor HLA A, B and DR for at least one allele. Four or five mismatches were present in 22 patients. However, upon analyzing HLA matches/mismatches in the eight patients who had acute rejection episodes (Table 3), no correlation between the occurrence of acute rejection and HLA mismatches was apparent.

**Table 2:** Frequency of HLA allele mismatches in all 49 patients

Number of HLA allele mismatches	Patients with HLA allele mismatches
1	4 (8.1%)
2	8 (16.3%)
3	15 (30.6%)
4	11 (22.4%)
5	11 (22.41%)
6	0

**Table 3:** HLA hetero and homozygous match/homozygous mismatch in the 8 patients who had acute rejection (AR) episodes

	HLA A	HLA B	HLA DR	Match/mismatch
Patient 1	Match	Match	Match	3/0
Patient 2	Match	Match	Match	3/0
Patient 3	Match	Match	Match	3/0
Patient 4	Match	Mismatch	Mismatch	1/2
Patient 5	Match	Match	Match	3/0
Patient 6	Match	Match	Match	3/0
Patient 7	Match	Mismatch	Match	2/1
Patient 8	Match	Match	Mismatch	2/1

### Acute rejection and SNPs

The number of acute rejection episodes in relation to the type of gene polymorphism (CYP3A5*3, CYP3A4B, ABCB1, SXR) is shown in Table 4. The twelve patients with A allele polymorphism for *CYP3A5* had a significantly higher number of acute rejection episodes as compared to the 37 with GG polymorphism (*p*-value < 0.05 at Fisher exact test). The nine patients homozygous GG for *SXR A7635G* polymorphism did not show any acute rejection episode, in contrast with the patients who had rejection episodes pertaining to the cohort of 40 carriers of A allele (*p*-value < 0.05). No significant correlation was found between *ABCB1* polymorphisms and rejection.

**Table 4:** Number of acute rejection episodes in relation to the different gene polymorphisms

GENE	polymorphism	N° of acute rejection episodes
**CYP3A5**	AG (*n* = 12) (24.4%)	5* (41.6%)
	GG (*n* = 37) (75.5%)	3 (1.0%)
**CYP3A4B**	AA (*n* = 43) (87.7%)	7 (16.2%)
	AG (*n* = 6) (12.2%)	1 (16.6%)
**SXR A7635G**	AA (*n* = 16) (32.6%)	2 (12.5%)
	AG (*n* = 24) (48.9%)	6 (25.0%)
	GG (*n* = 9) (18.3%)	0* (0.0%)
**SXR RS rs3842689**	In/in (*n* = 19) (38.7%)	3 (12.9%)
	In/del (*n* = 22) (44.8%)	4 (18.1%)
	Del/del (*n* = 8) (16.32%)	1 (12.5%)
**ABCB1 C1236T**	CC (*n* = 18) (36.7%)	3 (16.6%)
	CT (n = 19) (38.7%)	2 (10.5%)
	TT (n = 12) (24.4%)	3 (0.25%)
**ABCB1 G2677T/A**	GG (n = 16) (32.6%)	2 (12.5%)
	GT/A (*n* = 26) (53.0%)	5 (19.2%)
	TT (*n* = 7) (14.2%)	1 (14.2%)
**ABCB1 C3435T**	CC (*n* = 15) (30.6%)	4 (26.6%)
	CT (n = 24) (48.9%)	2 (8.3%)
	TT (*n* = 10) (20.4%)	2 (20.0%)

* *p*-value < 0.05 at Fisher exact test

### Drug trough level and genetic

Tacrolimus dose, blood trough levels and dose-normalized trough levels of the 49 patients from 6 to 360 days after transplantation are reported in Table 5 in relation to the different gene polymorphisms. Tacrolimus trough level (C0 normalized for dose/kg) of the 12 patients who were carriers of the A allele in CYP3A5*3 was significantly lower than that of the 37 who were not carriers (homozygous GG) throughout (*p*-value < 0.05 at Mann Whitney test for all considered time points). No differences were found in tacrolimus trough level of patients with all the other gene polymorphisms (data not shown).

**Table 5:** Tacrolimus pharmacokinetic data in relation to CYP3A5 and SXR A7635G gene polymorphisms in the 49 patients of the study

	Days	CYP3A5*3		SXR A7635G		
		AA/AG (12 patients)	GG (37 patients)	AA (16 patients)	AG (24 patients)	GG (9 patients)
Dose/kg (mg/kg)	day 7	0.17 ± 0.10	0.15 ± 0.06	0.18 ± 0.10	0.14 ± 0.04	0.16 ± 0.06
	day 30	0.18 ± 0.10	0.13 ± 0.06	0.17 ± 0.11	0.14 ± 0.05	0.14 ± 0.07
	day 90	0.15 ± 0.09	0.11 ± 0.06	0.14 ± 0.10	0.10 ± 0.05	0.12 ± 0.07
	day 180	0.13 ± 0.08	0.09 ± 0.06	0.11 ± 0.09	0.08 ± 0.04	0.11 ± 0.07
	day 360	0.11 ± 0.08	0.07 ± 0.05	0.10 ± 0.08	0.07 ± 0.03	0.08 ± 0.07
C0/(dose/kg) (ng/ml)/(mg/kg)	day 7	67.52 ± 48.67*	80.67 ± 58.46	95.47 ± 82.73	64.13 ± 24.06	79.11 ± 53.07
	day 30	73.83 ± 105.88*	111.45 ± 70.37	103.21 ± 87.98	97.40 ± 60.02	111.86 ± 118.66
	day 90	92.19 ± 139.61*	114.52 ± 68.18	103.33 ± 76.67	108.79 ± 64.90	119.26 ± 157.82
	day 180	70.48 ± 137.70*	137.70 ± 103.44	121.30 ± 101.09	127.20106.22	104.06 ± 70.10
	day 360	72.56 ± 182.92*	182.92 ± 199.75	192.34 ± 283.35	142.93 ± 103.30	121.24 ± 78.97

Data are expressed as mean ± S.D. * *p*-value < 0.05 at Mann Whitney test

Finally, considering the whole cohort of 49 patients (Table 6), no significant difference was present as regards tacrolimus trough levels between patients with acute rejection episodes and those without. Conversely, considering the eight patients with rejection episodes (Table 7), those with the A allele for CYP3A5*3 presented with a significantly lower tacrolimus trough level (*p*-value < 0.05 at Mann Whitney test) than those who were not carriers for A allele (homozygous GG). Moreover, the five patients with A allele for CYP3A5*3 who presented acute rejections episodes had a lower tacrolimus trough level in comparison to the seven who were carriers for allele A but did not show any acute rejection (*p*-value < 0.05 at Mann Whitney test).

**Table 6:** Tacrolimus pharmacokinetic data and occurrence of acute rejection (AR) in the 49 patients of the study

	Days	No AR (41 patients)	AR (8 patients)
Dose/kg (mg/kg)	day 7	0.17 ± 0.08	0.13 ± 0.03
	day 30	0.15 ± 0.08	0.15 ± 0.06
	day 90	0.11 ± 0.08	0.11 ± 0.08
	day 80	0.09 ± 0.07	0.10 ± 0.04
	day 360	0.07 ± 0.06	0.08 ± 0.04
C0/(dose/kg) (ng/ml)/(mg/kg)	day 7	78.07 ± 45.55	100.51 ± 89.31
	day 30	115.22 ± 86.70	72.48 ± 42.91
	day 90	115.37 ± 97.21	91.56 ± 49.57
	day 180	130.45 ± 102.76	90.36 ± 64.71
	day 360	190.11 ± 196.61	98.31 ± 51.3

Data are expressed as mean ± s.d. Mann Whitney was used as statistical test

**Table 7:** Tacrolimus pharmacokinetic data in relation to CYP3A5 gene polymorphisms in the 8 patients with acute rejection (AR)

	Days	AA/AG with AR (5 patients)	GG with AR (3 patients)	AA/AG without AR (7 patients)
Dose/kg (mg/kg)	day 7	0.14 ± 0.01	0.11 ± 0.05	0.19 ± 0.14
	day 30	0.20 ± 0.03*	0.09 ± 0.03	0.18 ± 0.13
	day 90	0.14 ± 0.05*	0.08 ± 0.01	0.16 ± 0.12
	day 80	0.13 ± 0.04*	0.06 ± 0.01	0.14 ± 0.10
	day 360	0.10 ± 0.04	0.05 ± 0.02	0.13 ± 0.10
C0/(dose/kg) (ng/ml)/(mg/kg)	day 7	55.56 ± 20.87	175.42 ± 120.49	74.92 ± 62.45
	day 30	45.81 ± 10.91 *#	105.81 ± 47.40	92.53 ± 49.62
	day 90	63.92 ± 26.57*#	126.11 ± 54.57	110.56 ± 55.25
	day 180	55.33 ± 23.42*	134.14 ± 77.98	79.78 ± 35.21
	day 360	79.48 ± 49.34	121.85 ± 50.87	66.35 ± 37.25

Data are expressed as mean ± s.d. Mann Whitney was used as statistical test

* *p*-value < 0.05 AA/AG vs GG patients with acute rejections

# *p*-value < 0.05 AA/Ag with acute rejection vs AA/AG without acute rejection

## Discussion

Several factors have been associated with the occurrence of acute rejection episodes during the first year after renal transplantation, namely the number of HLA mismatches, a low immunosuppressive drug blood concentration and, more recently, a series of gene polymorphisms [7, 28].

In our population, HLA mismatch did not seem to play a significant role in determining acute rejection rate (Table 3). Additionally, HLA mismatch had no significant role in the occurrence of acute rejection in a recent report by Parajuli et al., who analyzed 1102 kidney biopsies and did not find any correlation between the HLA mismatch and the risk of acute rejection [33].

The role of changes in drug metabolism, induced by polymorphisms of a number of genes, has been repeatedly underlined in the last two decades [11–19].

In particular, blood concentration of immunosuppressive drugs has a pivotal role in preventing acute rejection and allograft failure. Therapeutic tacrolimus blood concentration is particularly important during the first 3 months after transplantation [34] and a wide therapeutic window, from 5 to 9.5 ng/ml, is warranted during the first-year post transplantation [35]. To elucidate, the prescription of an adequate tacrolimus dose since the early post-transplant days is considered to be of the upmost importance [36]. Likewise, Hu et al., suggested a positive relation between the time needed to reach a therapeutic tacrolimus trough level and the occurrence of acute rejection. This relation is unique for each recipient and helps explain why in some cases acute rejection occurs despite tacrolimus being within the therapeutic range [37].

The importance of CYP3A5*3 gene polymorphism in affecting the bioavailability of tacrolimus, already suggested by our group [20], is confirmed by the pharmacokinetic data of this study (Tables 5 and 7). Our results also suggest that being a carrier of allele A for *CYP3A5* is not the only risk factor to be considered for the prevention of acute rejection, and that other factors may counterbalance its negative effect.

To explain further, the most interesting result of this study concerns the putative protective role of *SXR A7635G* homozygous GG polymorphism against acute rejection, which is the first report of a protective polymorphism in the immunology of kidney rejection.

Only a few studies about *SXR* gene polymorphism and rejection have been published so far. Two articles reported that subjects homozygous GG for *SXR A7635G* had an increase in the *CYP* and *ABCB1* expression [38, 39] and consequently, a low tacrolimus area under the curve [24–26]. In our study we did not find any positive correlation between the above cited *SXR* polymorphism and tacrolimus trough level (Table 5).

It can be argued that that the *SXR* protective effect does not result from an interference of the *SXR* polymorphism with the metabolism of tacrolimus, but rather from a possible suppression of the rejection mechanism itself, working upstream of the drug metabolic pathway [22, 23]. In fact, SXR makes a heterodymer with the protein HSP90, a chaperonine that is involved in acute rejection, and binds to FKBP5, a protein of the same family of the tacrolimus target protein [40–42]. Consequently, the interaction between SXR and HSP90- FKBP5 may interfere with the acute rejection mechanism.

According to our data, both pre-transplantation genetic screening for *SXR A7635G* and CYP3A5*3 polymorphisms and post-transplantation drug monitoring could help in preventing an ineffective tacrolimus trough level by identifying the carriers of either protective or risk factors.

A limitation of this study is the relatively low number of patients for each evaluated cohort, in particular of patients GG for *SXR A7635G*. However, these numbers are similar to those of other pediatric articles on the same topic [43, 44].

## Conclusion

In conclusion, this study, along with the other retrospective studies [45, 46], demostrate the importance of pharmacokinetics and pharmacogenomics to decrease the occurrence of acute rejection, however, there still remain several barriers to their routine clinical application [46]. Pharmacogenomics of tacrolimus can drive the clinical decision regarding the starting dose, with a benefit for the transplanted patients, as it was previously described [46]. Even if pharmacogenetics suffer from some grade of imprecision, due to the interaction of various polymorphisms, in the case of tacrolimus it should be performed together with the classical therapeutic drug monitoring, which is able to reduce the inter-individual pharmacokinetic variability.

In this way, the genetic analysis for CYP3A5*3 and *SXR A7635G* polymorphisms, performed in advance of transplantation, may be of help in forecasting the risk of acute rejection and in choosing the appropriate tacrolimus dosage for each individual patient in the first year after kidney transplantation.

## Data Availability

The datasets during and/or analysed during the current study available from the corresponding author on reasonable request.

## Abbreviations

SNP: Single nucleotide polymorphism
HLA: Human leucocyte antigen
CYP: Cytochrome P-450
CYP3A5: Cytochrome P-450 3A5
CYP3A5*3: Variant *3 of cytochrome P-450 3A5 rs776746:G > A
SXR: Steroid xenobiotic receptor
ABCB 1: ATP Binding Cassette Subfamily B Member 1
HSP90: Heat shock protein 90
FKBP: FK506 binding protein
